# Alanine Scanning of Cucumber Mosaic Virus (CMV) 2B Protein Identifies Different Positions for Cell-To-Cell Movement and Gene Silencing Suppressor Activity

**DOI:** 10.1371/journal.pone.0112095

**Published:** 2014-11-07

**Authors:** Katalin Nemes, Ákos Gellért, Ervin Balázs, Katalin Salánki

**Affiliations:** 1 Plant Protection Institute, Centre for Agricultural Research, Hungarian Academy of Sciences, Budapest, Hungary; 2 Department of Plant Pathology, Corvinus University of Budapest, Budapest, Hungary; 3 Agricultural Institute, Centre for Agricultural Research, Hungarian Academy of Sciences Department of Applied Genomics, Martonvásár, Hungary; 4 Agricultural Biotechnology Center, Gödöllő, Hungary; Washington State University, United States of America

## Abstract

The multifunctional 2b protein of CMV has a role in the long distance and local movement of the virus, in symptom formation, in evasion of defense mediated by salicylic acid as well as in suppression of RNA silencing. The role of conserved amino acid sequence domains were analyzed previously in the protein function, but comprehensive analysis of this protein was not carried out until recently. We have analyzed all over the 2b protein by alanine scanning mutagenesis changing three consecutive amino acids (aa) to alanine. We have identified eight aa triplets as key determinants of the 2b protein function in virus infection. Four of them (KKQ/22-24/AAA, QNR/31-33/AAA, RER/34-36/AAA, SPS/40-42/AAA) overlap with previously determined regions indispensable in gene silencing suppressor function. We have identified two additional triplets necessary for the suppressor function of the 2b protein (LPF/55-57/AAA, NVE/10-12/AAA), and two other positions were required for cell-to-cell movement of the virus (MEL/1-3/AAA, RHV/70-72/AAA), which are not essential for suppressor activity.

## Introduction

The genome of plant viruses is quite limited coding only a few genes. In consequence each gene has multiple functions. For example the genome of *Cucumber mosaic virus* (CMV) belonging to the *Cucumovirus* genus codes only five proteins and among them the smallest one is the 2b protein which has roles in symptom induction [1], virus movement and evasion of the defense mechanism mediated by salicylic acid [2] [3] and jasmonic acid [4]. The 2b protein could also suppress the antiviral RNA silencing; it was among the first viral proteins described as an RNA silencing suppressor [5].

RNA silencing mediated by short-interfering RNAs (siRNAs) is a potent antiviral defense mechanism, and many plant viruses encode viral suppressors of RNA silencing (VSRs), although there is great diversity in the mode of action [6]. 2b protein is unique among the known plant and animal VSRs because it directly interacts with both the RNA and protein components of the RNA silencing machinery [7]–[11]. The 2b protein of CMV and *Tomato aspermy virus* (TAV), which also belongs to the *Cucumovirus* genus binds duplex siRNA *in vitro* [8]–[10] and TAV 2b proteins form dimers as it was demonstrated previously by immunoblot analysis in infected plants [12]. The crystal structure of the N-terminal 69 amino acids of TAV 2b, which region has a highly conserved amino acid (aa) sequence among cucumoviral 2b proteins [13], forms a dsRNA binding domain folded into two long helices connected by a short linker [8] [14]. The siRNA binding has a similar mechanism as it was described in the case of *Carnation Italian ringspot virus* P19 [15]. The length of siRNA duplexes is measured by a pair of hook-like structures that depend on a Trp residue (Trp-50) of the C-terminal-helix, which, however, is not conserved in other cucumoviral 2b proteins [13] [14]. The 2b protein of CMV is active *in vivo* to suppress the RNA-dependent RNA polymerase 6 (RDR6) dependent RNA silencing that targets both the infecting CMV and the transgenes either in stable transgenic plants or delivered transiently by *Agrobacterium tumefaciens* coinfiltration in *Nicotiana benthamiana* [16]–[18]. 2b binds ARGONAUTE1 (AGO1) [9] [7], which is an RNA ‘slicer’ enzyme known to be involved in plant antiviral RNA silencing [19] [20]. The interaction of CMV 2b and ARGONAUTE4 (AGO4) from *Arabidopsis* has been demonstrated *in vitro* and *in vivo* by co-immunoprecipitation and bimolecular fluorescence complementation assays, which are consistent with the observed activity of CMV 2b to suppress the *in vitro* slicer activity of AGO4 [9] [10]. Intriguingly, although the positive-strand RNA genome of CMV replicates exclusively in the cytoplasm, 2b is predominantly localized to the nucleus by single or double nuclear localization signals (NLSs) in subgroup II and I strains of CMV, respectively [21] [22].

The 2b proteins of different CMV strains and other cucumoviruses share a number of conserved amino acid sequence motives, suggesting important roles in protein functions. A number of these motives were analyzed previously and different functional domains were identified and characterized like nuclear localization signals (NLS), RNA binding domain (overlapping the NLSs), putative phosphorylation sites, as well as the N and C termini (involved in DNA binding) [23] [21] [24] [25]. Since systematic analysis of the 2b protein was not carried out previously, we analyzed the effect of mutations entirely along the 2b protein in the viral infection cycle.

## Results

### Construction the alanine scanning mutants of the 2b protein

**Figure 1:**
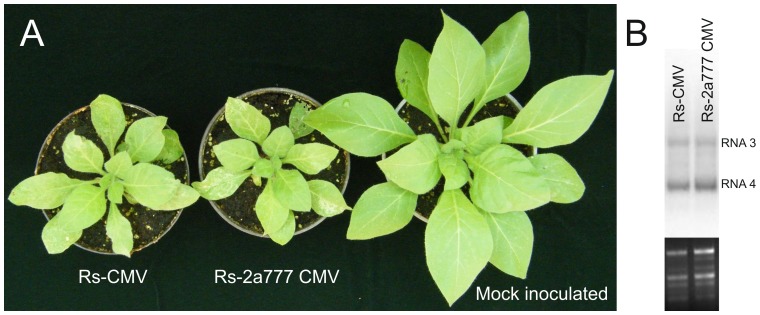
Symptoms elicited 14 days after the inoculation on *Nicotiana clevelandii* plants by the Rs-CMV and mutated Rs2-2a777 CMV virus (A). Northern blot hybridization analysis of total RNAs extracted from non-inoculated leaves 6 weeks after inoculation (B). The radiolabeled probe was specific for Rs-CMV RNA3. Ethidium bromide-stained rRNA from the same volume of each sample is shown below each lane.

Alanine scanning is simple and widely used technique determining the functional role of protein residues [26]. We intended to replace three consecutive amino acids of CMV 2b protein to alanine. Since the carboxy terminal region of the 2a protein overlaps with the amino terminal part of the 2b protein, first a STOP codon was introduced into the infectious clone of RNA2 into the 2a protein ORF just preceding the start codon of 2b protein. The resulting clone (Rs2-2a777 CMV) coded for a truncated 2a protein missing the 80 carboxy terminal aas and a full length 2b protein. The infectivity and the stability of the mutant transcript in the presence of the wild type RNA 1 and 3 was monitored on *Nicotiana clevelandii* plants by RT/PCR and nucleotide sequence determination for a six week period after infection. The mutation retained during this period, and no alteration of the symptom phenotype has been observed between Rs2-2a777 and the wild-type virus (Rs). The Northern analysis demonstrated that the viral RNA accumulation was not distinct from the wild type virus (Fig. 1A, B). These results proved that the carboxy terminal 80 amino acids of the 2a protein can be deleted without changing the infection phenotype on this host. For construction the alanine scanning mutants we used the pRs2-2a777 clone. Altogether 37 mutants were constructed replacing the three consecutive aas of the 2b protein by alanine. Name of the constructs indicate the original amino acids and the position of the exchange in the 2b protein sequence (for example MEL/1-3/AAA, NVG/4-6/AAA, etc.).

### *In vivo* characterization of 2b protein mutants

The wild-type (WT: Rs2-2a777) and mutated RNA2 *in vitro* transcripts were combined as appropriate with *in vitro* synthesized Rs-CMV RNAs 1 and 3 transcripts for inoculation of *Nicotiana clevelandii* and *Chenopodium murale* plants. The development of symptoms was monitored for thirty days period after the inoculation.

**Figure 2:**
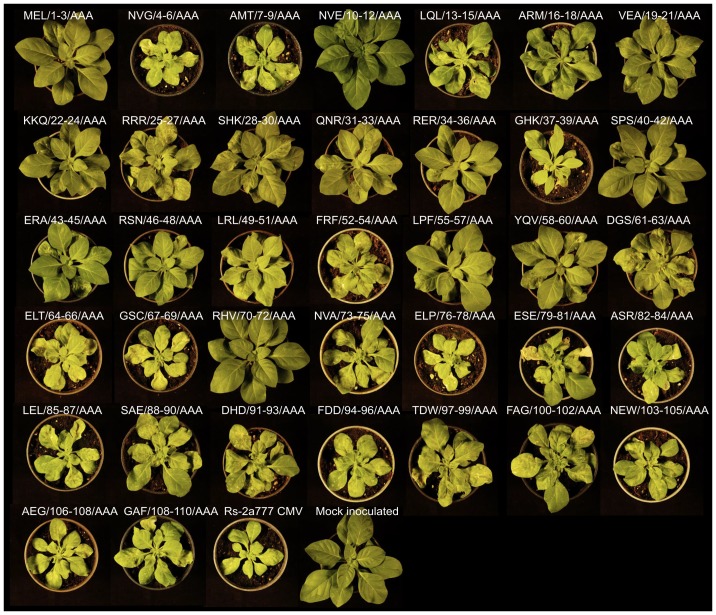
Symptoms elicited 14 days after the inoculation on *Nicotiana clevelandii* plants by the Rs-2a777 CMV and the alanine-scanning mutated Rs2-CMV viruses. Name of the pictures taken from the plants indicate the consecutive amino acids of the exchange in the 2b protein sequence.

The majority of the mutant viruses caused similar symptoms as the original Rs-CMV on *Nicotiana clevelandii* (Fig. 2). In four cases symptoms were not emerged during the thirty days of the monitoring period (MEL/1-3/AAA, NVE/10-12/AAA, SPS/40-42/AAA, HRV/70-72/AAA), and in the case of four further constructs (KKQ/22-24/AAA, QNR/31-33/AAA, RER/34-36/AAA, LPF/55-57/AAA) the symptoms were much milder compared to the wild type virus (Rs2-2a777) (Fig. 2). Among these mutants in six cases the virion could be purified thirty days after the inoculation (NVE/10-12/AAA, KKQ/22-24/AAA, QNR/31-33/AAA, RER/34-36/AAA, SPS/40-42/AAA, LPF/55-57/AAA) but the virus yield was significantly lower than in the case of the other mutants and the wild type virus (data not shown).

**Figure 3:**
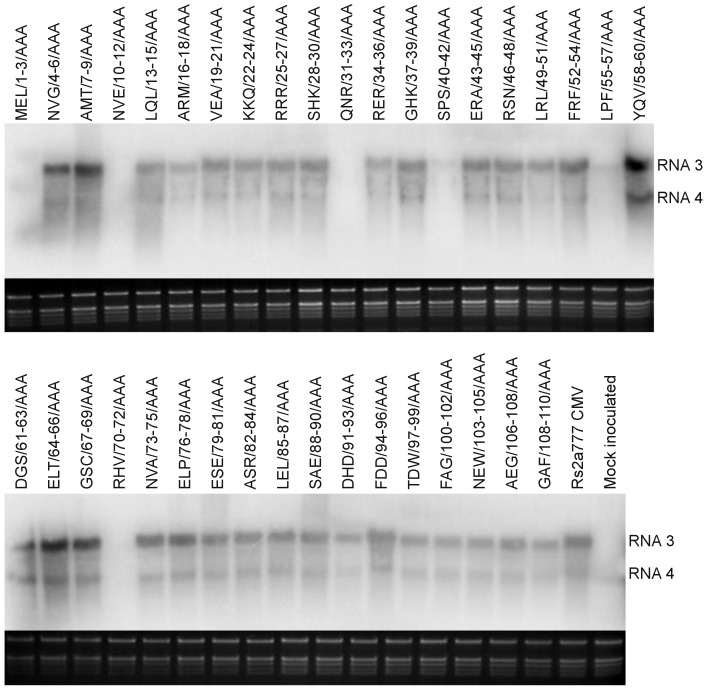
Northern blot analysis of *Nicotiana clevelandii* plants 8 days after inoculation. Total RNAs were extracted from non-inoculated leaves. The radiolabeled probe was specific for Rs-CMV RNA3. Ethidium bromide-stained rRNA from the same volume of each sample is shown below each lane.

**Figure 4:**
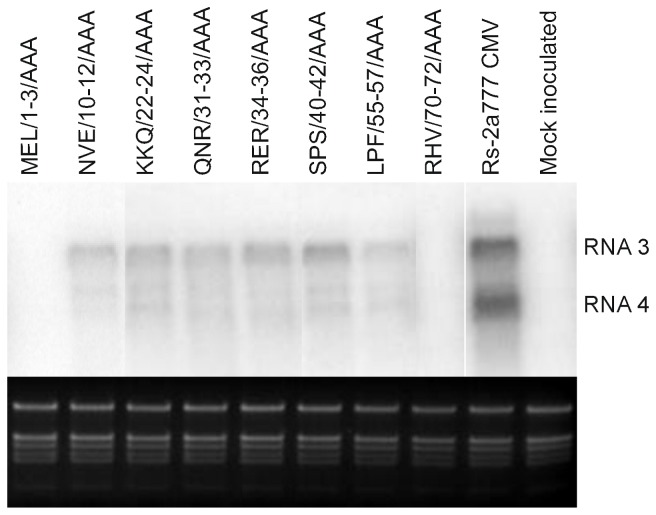
Northern blot analysis of *Nicotiana clevelandii* plants 30 days after inoculation with Rs-CMV, and with the eight mutant caused altered phenotype on *N. clevelandii* plants. Total RNAs were extracted from non-inoculated leaves. The radiolabeled probe was specific for Rs-CMV RNA3. Ethidium bromide-stained rRNA from the same volume of each sample is shown below each lane.

Eight days after inoculation the viral RNA was detectable (Fig. 3) in the non inoculated leaves of the infected plant at the great majority of the different constructs even if the viral RNA concentration was greatly reduced in two cases (SPS/40-42/AAA, LPF/55-57/AAA) and viral RNA was not detectable at four further mutants (MEL/1-3/AAA, NVE/10-12/AAA, QNR/31-33/AAA, HRV/70-72/AAA). Thirty days after inoculation the viral RNA was detectable at six mutants showing no or modulate symptoms, but the amount of the viral RNA was still significantly reduced. The Northern analyses of these plants elucidate the low efficiency of virus purification of these mutants (Fig. 4). We could never detect the presence of MEL/1-3/AAA and RHV/70-72/AAA in non infected leaves during thirty days of the experiment in five independent experiments. The identity of all the mutants was confirmed by RT/PCR nucleotide sequence determination from the systematically infected leaves.

**Figure 5:**
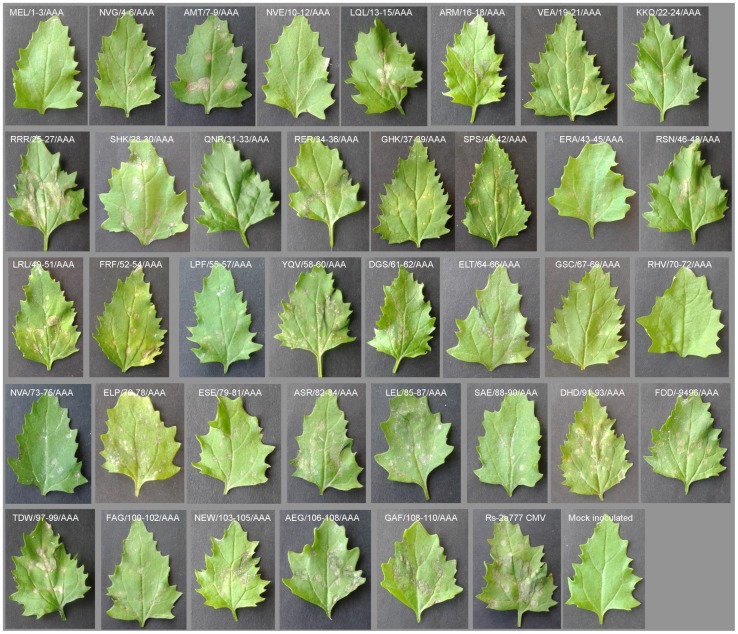
Symptoms elicited 5 days after the inoculation on *Chenopodium murale* plants inoculated by the Rs-2a777 CMV and the alanine-scanning mutant Rs2-CMV transcripts in the present of RNA1, 3 transcripts of Rs-CMV. The name of the constructs is indicated on the pictures.

The majority of the mutant viruses caused local lesions on *Chenopodium murale* as the wild-type virus (WT: Rs2-2a777) although the phenotype of the local lesions were diverse. In the case of mutant MEL/1-3/AAA and mutant HRV/70-72/AAA local lesions were not (Fig. 5).

### Gene silencing suppressor activity of the symptom modulated mutants

Since the primary function of the CMV 2b protein is the gene silencing suppressor activity, we have analyzed this in the case of the eight mutants bearing altered phenotype in the previous experiment using *Agrobacterium*-mediated transient assay. Binary vector expressing GFP reporter gene was agroinfiltrated into transgenic *Nicotiana benthamiana* (silenced for GFP expression) leaves together with the binary vector expressing the wild type 2b protein or the mutant ones (MEL/1-3/AAA, NVE/10-12/AAA, SPS/40-42/AAA, KKQ/22-24/AAA, QNR/31-33/AAA, RER/34-36/AAA, LPF/55-57/AAA, RHV/70-72/AAA). The suppressor activities were monitored by visual observation of the GFP fluorescence and quantitatively by measuring the accumulation level of GFP RNA in the infiltrated leaves by qRT-PCR.

**Figure 6:**
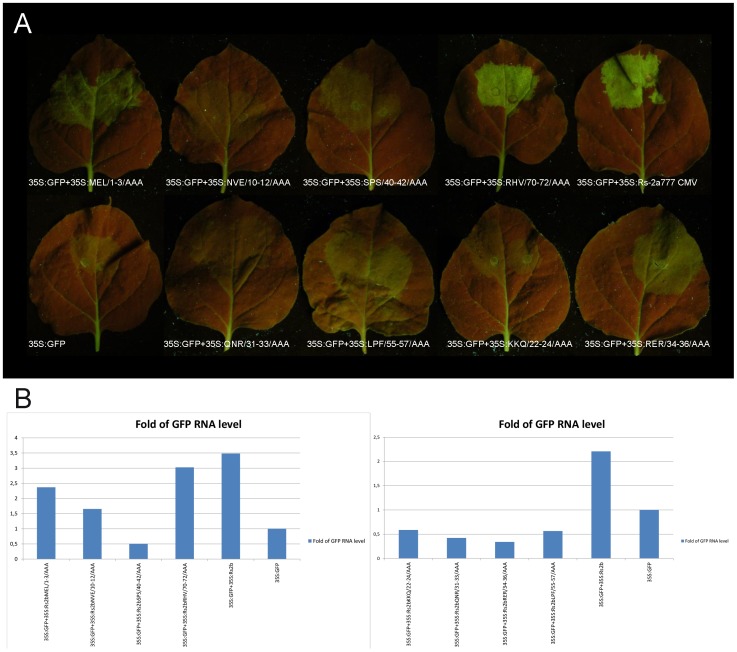
Suppression of RNA silencing in patch assays. A binary vector expressing the GFP reporter gene was agroinfiltred into *Nicotiana benthamiana* leaves, together with an empty binary vector or together with binary vectors expressing 2b protein and MEL/1-3/AAA, NVE/10-12/AAA, SPS/40-42/AAA, RHV/70-72/AAA, KKQ/22-24/AAA, QNR/31-33/AAA, RER/34-36/AAA and LPF/55-57/AAA 2b protein construct.

The visual observation revealed that at six out of the eight mutants the GFP fluorescence is greatly reduced (NVE/10-12/AAA, SPS/40-42/AAA, KKQ/22-24/AAA, QNR/31-33/AAA, RER/34-36/AAA, LPF/55-57/AAA). In one case (MEL/1-3/AAA) the fluorescence is slightly weaker compared to the wild type 2b mutant and in the case of RHV/70-72/AAA mutant the fluorescence is hardly affected (Fig. 6A).

GFP mRNA levels in the presence of the suppressors were determined by qRT-PCR. The level of the *Nicotiana benthamiana* EF1α transcript was used as a normalization control. The qRT-PCR study confirmed the visual observation, proving the extreme reduction of the fold of GFP RNA level in the case of the mutants SPS/40-42/AAA, KKQ/22-24/AAA, QNR/31-33/AAA, RER/34-36/AAA and LPF/55-57/AAA. In the case of NVE/10-12/AAA the reduction is about half of the expression of the wild type construct, while at the MEL/1-3/AAA and RHV/70-72/AAA mutants the reduction is substantially smaller. In these cases the constructs were still able to suppress efficiently the partial silencing of the GFP reporter gene, increasing the levels of the GFP-derived green fluorescence. In case of constructs NVE/10-12/AAA, SPS/40-42/AAA, KKQ/22-24/AAA, QNR/31-33/AAA, RER/34-36/AAA and LPF/55-57/AAA decreased levels of green fluorescence have proved the defense of gene silencing suppressor activity of the mutated 2b proteins (Fig. 6B).

Excluding the role of the 2b protein stability in the previous experiments, the accumulation of the eight two 2b mutants have been analyzed by western blot in the infiltrated patches. We added six histidine residues to the C terminus of the 2b protein (Rs2a777) to create Rs2a777His similarly to Du et al., 2014. Rs2a777 and Rs2a777His were transiently expressed in *N. benthamiana* by agroinfiltration. The visual observation and qRT-PCR showed that the fluorescence was at the same level in the case of Rs2a777 and Rs2a777His and the Western blot showed equivalent accumulation of green fluorescent protein suggested that the silencing suppressor activities are at the same level which is also coincident with a previous study [27] (data not shown).

**Figure 7:**
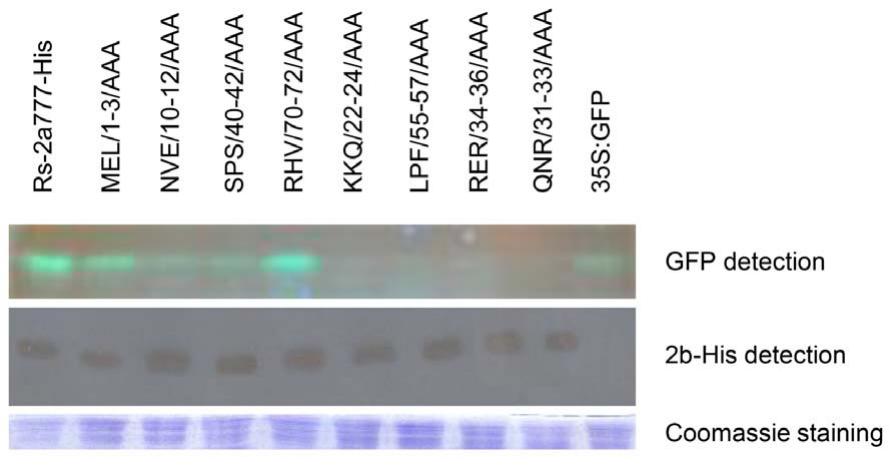
Immunoblot analyses of accumulation His-tagged 2b protein mutants in agroinfiltrated patches. Detection of the fluorescence of GFP proteins on SDS-PAGE by illuminating the gel with UV lamp. A penta-his antibody was detection of His-tagged 2b proteins. Coomassie staining was used to monitor the equivalence of protein loading and transfer.

Since the histidine tagging caused no reduction in the silencing suppressor activity of the Rs2b protein, we added histidine residues to the eight mutants bearing altered phenotypes. The histidine tagged mutants were transiently expressed in *N. benthamiana* by agroinfiltration. The accumulation of the mutant proteins were analyzed by western-blot (Fig. 7) indicating that the different GFP levels caused by the different suppressor activities not by the instability of the proteins. Taken together, all these data suggest that mutants NVE/10-12/AAA, SPS/40-42/AAA, KKQ/22-24/AAA, QNR/31-33/AAA, RER/34-36/AAA and LPF/55-57/AAA are less efficient inhibitors of local RNA-silencing than the wild-type 2b protein, while the suppressor affinity of the MEL/1-3/AAA and RHV/70-72/AAA mutants is hardy affected.

### Analysis of the cell-to-cell movement of the symptom modulated mutants

**Figure 8:**
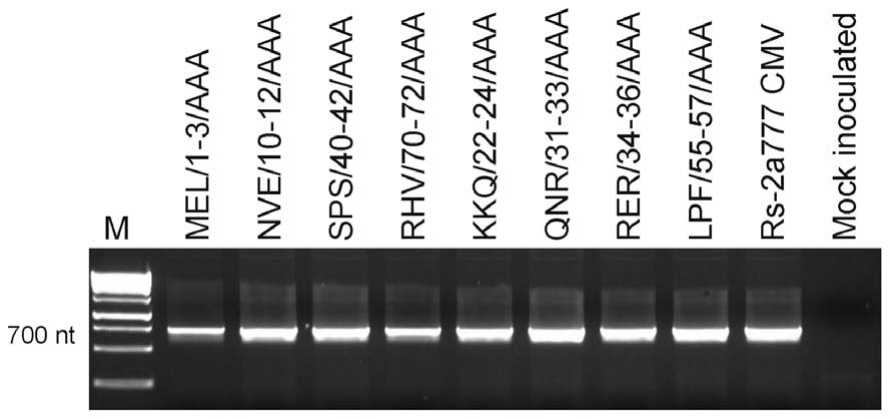
Detection of Rs-CMV and the eight mutants in inoculated leaves of *Nicotiana clevelandii* 3 days after inoculation. Samples were analyzed by RT-PCR using primers specific for RNA2 of CMV. M, DNA molecular size marker, 1000 bp and 1500 bp makers are indicated.

Since the analysis of the gene silencing suppressor activity of the mutants with altered phenotype does not explain the symptom modulation in all cases, the cell-to-cell movement of the mutants was investigated. First RT-PCR was carried out from inoculated leaves of *Nicotiana clevelandii* 3 days after inoculation. All of the eight mutants could be detected 3 days after inoculation (Fig. 8).

**Figure 9:**
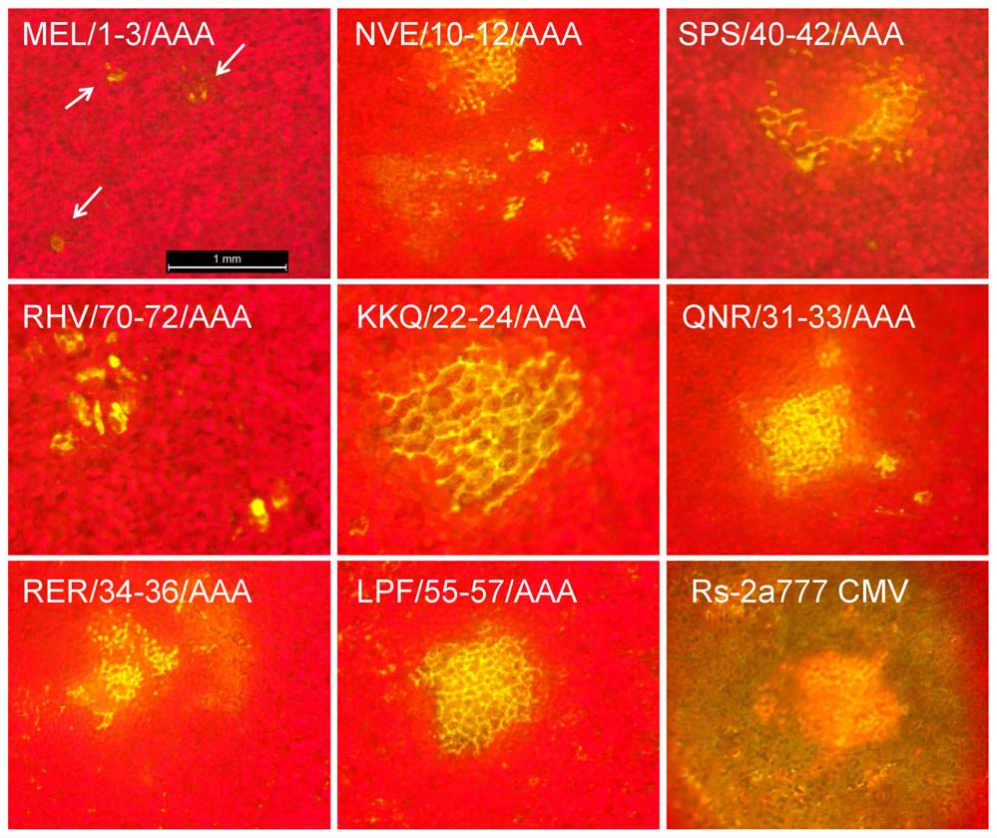
Development of fluorescence in *Chenopodium murale* plants infected with GPF-expressing derivatives of Rs-CMV and the eight mutant (MEL/1-3/AAA, NVE/10-12/AAA, SPS/40-42/AAA, RHV/70-72/AAA, KKQ/22-24/AAA, QNR/31-33/AAA, RER/34-36/AAA and Rs2LPF/55-57/AAA) constructs. The red background is chlorophyll fluorescence from intact epidermal and mesophyll cells. Green-yellow represents the GFP-derived fluorescence from the chimera virus and the autofluorescence from necrotic tissue. The arrows point at the single epidermal GFP-fluorescent cells.

In a former work of our group a recombinant RNA 3 molecule was constructed to follow the virus movement visually [28]. The CP was replaced with GFP gene and the movement protein of CMV was exchanged with the MP of *Cymbidium ringspot virus* (CMVcymMPΔCP-GFP). Local movement of this construct can be visualized by epifluorescence microscopy observing development of fluorescent foci in *Chenopodium* species. Using *in vitro* transcripts of pCMVcymMPΔCP-GFP, pRs1 and either of the eight mutants causing altered symptoms, *Chenopodium murale* plants were infected. Spreading of virus mutants NVE/10-12/AAA, SPS/40-42/AAA, KKQ/22-24/AAA, QNR/31-33/AAA, RER/34-36/AAA and LPF/55-57/AAA was clearly visible under UV illumination epifluorescence microscopy and proved that GFP expression was not confined to the initially infected cells, and the virus efficiently spread from the primary infected cell to the neighboring ones. On the plant leaves infected with mutant MEL/1-3/AAA and RHV/70-72/AAA, only numerous isolated infected cells were detected, so infection was restricted to the single infected cells even 3 days after inoculation (Fig. 9).

## Discussion

In the present study the systematic analysis of the 2b protein of CMV has been carried out by the means of alanine-scanning mutagenesis. According to our results eight out of the 37 mutants has dramatic effect on the infectivity of CMV on *Nicotiana clevelandii* plants. As the 2b protein of CMV is a multifunctional protein, which is involved in nearly all steps of the virus infection cycle and also in suppression of the RNAi-mediated defense mechanism of plant, the majority of the defective mutants were damaged in the RNA silencing suppressor activity.

The RNA silencing composes the primary plant immune system against viruses. Antiviral RNA silencing is triggered by dsRNA replication intermediates or intramolecular fold-back structures within viral genomes [29], [30]. These viral dsRNAs are mainly processed by Dicer-like protein 4 (DCL4) or its surrogate Dicer-like protein 2 (DCL2), to produce 21- or 22-nt virus-derived small RNAs (vsRNAs), respectively [31], [32]. vsRNAs are subsequently recruited, mainly by AGO1 and AGO2, to direct PTGS of viral RNA as part of antiviral RISCs [33] [34] [7]. To counteract this defense mechanism, plant viruses produce different suppressors of RNA silencing (VSRs). The CMV 2b protein was one of the first VSRs shown to interact physically with AGO1, and this interaction leads to inhibition of AGO1 slicing activity in a RISC *in vitro* reconstituted assay [7]. 2b protein has been also shown to bind siRNA *in vitro* [9]. Expressing 2b protein prevents the spread of the systemic silencing signal in tissues and consequently the induction of silencing in target cells [35]. Binding of siRNA is crucial for the 2b protein silencing suppressor activity and according to recent results the suppressor activity is independent of AGO binding [11].

Four of the mutants with defective gene silencing suppressor activity are localized in previously identified functionally essential regions of the 2b protein. The region where the KKQ/22-24/AAA, the QNR/31-33/AAA and the RER/34-36/AAA localized was proved to participate in sRNA binding [14] [9] [36] and if this region is deleted, the gene silencing suppressor function is damaged.

The position of the mutations in three cases (KKQ/22-24/AAA, QNR/31-33/AAA, RER/34-36/AAA) overlap with nuclear localization signals, which sites are highly conserved in all CMV isolates [21] [23] and deletion of these sites led to cytoplasmic localization of the protein [11]. Recently it was proved that nuclear localization is not required for gene silencing suppressor activity [11]. The infection properties of the RRR/25-27/AAA mutant also confirm that the nuclear localization signal can be modified without altering the infection phenotype of the CMV.

**Figure 10:**
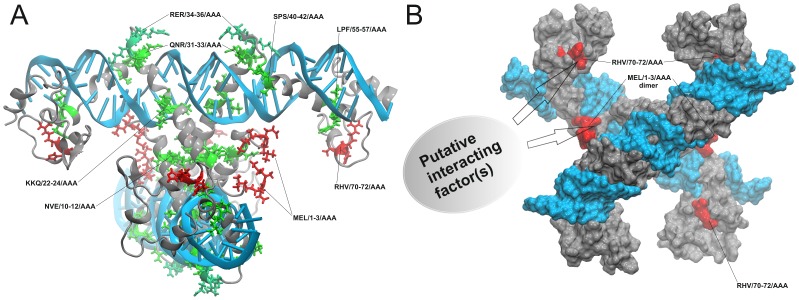
Cartoon representation of the siRNA-2b nucleoprotein complex with the localization of those eight mutants whose bearing altered phenotype on *Nicotiana clevelandii* plants. Aa triplets which are involved in the putative cell-to-cell movement related interactions are red while the other six mutations are colored green (A). Molecular surface representation of the siRNA-2b nucleoprotein complex. siRNA surfaces are colored light blue while 2b protein subunits are grey. Red protein surface regions indicate the three-dimensional localizations of those mutations which lead to movement-deficient behavior (B).

The crystal structure of the homologous truncated 2b protein of TAV has been determined in 2008 [14]. The determined part of the 2b protein contains two long alpha-helices. The helical axes rotate 120° angle to each other. The 2b protein forms a pair of hook-like dimers to bind siRNA duplex. The alpha-helices fit into the major groove of the siRNA in a length-preference and sequence-independent manner. The biologically active form is tetramer: four 2b protein molecules bind two siRNA duplexes. The C-terminal domain (aa 69–110) of 2b protein is missing from the X-ray structure therefore a reliable, full-length Rs-CMV 2b protein model was generated with molecular modeling methods [37] [14]. The active siRNA bound tetramer form was also constructed. Since the mutations in the KKQ/22-24/AAA, QNR/31-33/AAA, RER/34-36/AAA mutants localize in the middle and at the end of the first α-helix in the RNA binding surface of the protein, presumable the inadequate RNA binding induces the functional defect of these modified proteins (Fig. 10A). According to our study most likely the less effective suppression of local gene silencing is a result of the damaged structure of these mutants and not the absence of nuclear localization.

In the case of SPS/40–42/AAA which was also asymptomatic on *Nicotiana clevelandii* plant and showed reduced gene silencing suppressor activity in patch assay the mutations located in the putative phosphorylation site [21]. This phosphorylation site is conserved in all of the CMV isolates, and previously described essential for nuclear accumulation and siRNAs binding to suppress PTGS [8] [9]. Both serines were found to be required for symptom induction [38]. This mutation is located in the forepart of the second α-helix. Most likely this mutation disrupts the integrity of the second α-helix and presumably silencing suppressor activity decreases due to the sake of the protein structure.

In the case of the NVE/10-12/AAA and LPF/55-57/AAA mutants the infectivity of the virus and the PTGS suppressor activity reduced remarkably, but these positions of the 2b protein were not analyzed in previous studies. NVE/10-12/AAA localizes in the forepart of the first α-helix, which is involved in the leucine-zipper-like tetramerization mechanism. Our *in silico* analysis suggests that this mutation does not allow the formation of the active tetrameric structure (Fig. 10A). This mutant has retained partially the gene silencing suppressor activity but it was marginally lower compared to the wild type according to the qRT-PCR results. LPF/55-57/AAA is located in the end of the second α-helix. These residues immersed into the mayor groove of the siRNA complex. The experimental data suggest that in the case of these constructions evolve very slowly. Based on the tetramer structure it can be rendered probable, that these mutations produce reduced stability siRNA-protein complexes without losing its functionality (Fig. 10A). The reduction of the gene silencing suppressor activity of the previously discussed mutants does not prevent the cell-to-cell movement as the GFP fluorescence indicates using GFP labeled RNA molecules, but the virus concentration was significantly lower compared to the wild-type virus.

Beside binding siRNAs, 2b proteins could interact with different host proteins such as AGO1, AGO4 and catalase 3. These interactions lead to different levels of the viral pathogenicity and virulence. 2b protein also has been shown to be involved in local and systemic movement of the virus, although the role of it is poorly understood. A mutant of the subgroup II CMV strain Q which cannot express the 2b protein was unable to move systemically in cucumber and displayed decreased symptom induction on *Nicotiana glutinosa* and on *Nicotiana tabacum*, which results suggest the role of 2b protein in viral systemic movement [39] [40]. Deletion or interruption of the 2b ORF generally results in less efficient or altered local movement of CMV [40] [41], cucumovirus reassortants [41] and peanut stunt virus [42]. But in these cases the indirect role of 2b protein through RNAi suppression in the altered viral movement was not excluded. Binding of short RNAs correlates with RNA silencing suppression activity of the 2b protein [36]. In the case of two mutants (MEL/1–3/AAA and RHV/70–72/AAA) the gene silencing suppressor activity have not changed significantly according to the patch assay and qRT-PCR results, but the virus localized in single infected cells, and systematic infection never was detected neither symptoms were observed. MEL/1-3/AAA and RHV/70–72/AAA in patch assay were able to suppress efficiently the partial silencing of GFP (Fig. 6A), and gene silencing suppressor activity was only slightly reduced compared to the wild-type 2b protein according to the qRT-PCR results (Fig. 6B). In infectivity assay using GFP expressing RNA 3 recombinants on *Chenopodium murale*, we could detect GFP fluorescence only in a few single cells, so our analysis demonstrates that these sites are substantial for the local movement of the virus. These results directly prove that the 2b protein has a function in the viral cell-to-cell movement independently of the gene silencing suppressor activity. Both the aa region 1–3 and 70–72 are strictly conserved in subgroup I CMV isolates. At the subgroup II isolates the aa 1–3 is also conserved, but the 70–72 aa region is located in the nine aa long regions missing from these isolates. Previously the requirement of N-terminal 17 aa was demonstrated in symptom induction but the virus was not localized to single cells [1]. Regarding to the 2b protein structure the first three residues of the 2b protein are in the centre of the siRNA bound tetramer but these amino acid side chains did not take part in the leucine-zipper-like α-helix connections. These first two or three residues are missing from the X-ray structure of the homologous TAV 2b tetramer [14] because of their disordered nature. On the basis of structural considerations we can conclude that the first three amino acids of the 2b protein are involved in a cell-to-cell movement related biomolecular interaction (Fig. 10B). The same conclusion could be drawn in the case of the other movement-deficient construct RHV/70-72/AAA. However, the X-ray structure of this part of the 2b protein is unknown and only molecular modeling results are available from the C-terminal domain of the CMV 2b protein [37]. Structural observation derived from molecular dynamics (MD) simulation of this C-terminal domain shows that this short protein sequence part (70 to 72) is located in a small α-helix. The His71 side chain is in solvent exposed position, which can play a significant role in a protein-protein interaction in the mechanism of the cell-to-cell movement (Fig. 10B). This is the first report demonstrating that the CMV 2b protein has a direct role in the local virus movement independently of its gene silencing suppressor activity.

## Materials and Methods

### Plasmid constructions

Description of the Rs-CMV and the infectious transcripts (pRs1, pRs2, pRs3) has been published previously [43]. A STOP codon was introduced into pRs2 into the 2a protein ORF just preceding the start codon of the 2b protein by PCR directed mutagenesis (pRs2-2a777) using the following oligonucleotides: 5′-CGTTGAGCTCCAT**ATT**ACTTTCGCTGTTTGTTGG-3′ (reverse), 5′-TATGGAGCTCAACGTAGGTGCAATGACAAACG-3′ (forward). Mutated nucleotides are in bold and the SacI restriction site is underlined.

Alanine scanning mutants of 2b protein were generated using the pRs2-2a777 clone by PCR directed mutagenesis. First the 2133–3052 fragment of this clone was subcloned into pGEM-T-easy vector and after mutagenesis and nucleotide sequence confirmation the 2133–3052 fragments of the proper clones were subcloned back to the pRS2-2a777. The sequences of primers used are detailed in Table S1. The restriction site (PstI) is underlined and the mutated nucleotides are written in bold.

### Test plants and plant inoculation

*Nicotiana clevelandii* Gray and *Chenopodium murale* plants were mechanically inoculated with wild type and *in vitro* mutated RNA2 transcripts in the presence of wild type RNA1 and RNA3 transcripts when the plants were at four-to-five leaf stage. Plants were maintained under normal glasshouse conditions (with a cycle of 14 h of light (22°C) and 10 h of dark (18°C).

### Analysis of plants

Total RNA was extracted from 200 mg systemically infected leaves 4 and 8 days after inoculation [44]. Virus RNA accumulation was followed by Northern blot analysis. Approximately 100 ng total RNA was denatured with formaldehyde and separated in formamide-containing agarose gels and blotted on to nylon membranes [45]. Northern blot hybridization analysis was performed with random-primed 32P-labelled DNA fragments specific for the Rs-CMV RNA3 sequence.

RT-PCR/DNA sequence determination was performed to analyze the stability of the mutant viruses with the Qiagen OneStep RT-PCR kit according to the manufacturer's instructions, using primers flanking of 2b coding region (forward 5′-GTTTGCCTGGTGTTACGACACCGA -3′, reverse 5′-GCGGATCCTGGTCTCCTTTTGGAGGCCC-3′). PCR products were purified by High Pure PCR product Purification Kit (Roche) prior nucleotide sequence determination.

### *Agrobacterium* infiltration

*Nicotiana benthamiana* GFP transgenic line 16c way kindly provided by Dr. Dániel Silhavy. *Agrobacterium*-mediated transient expression on *Nicotiana benthamiana* leaves was conducted by pressure infiltration as described previously [46] [47]. *Agrobacterium* culture of GFP-expressing strain was adjusted to a final optical density at 600 nm (OD_600_) 0.4 and the strains expressing the various 2b mutants to 0.2.

### GFP imaging

For visually detection of GFP fluorescence patches on leaves and with PAGE, a Blak-Ray B-100SP UV lamp (UVP) was used, and images were taken with Nikon D100 digital camera mounted with yellow lens (Hama HTMC filter).

For visually detection of GFP fluorescence of local movement Leica MZ10F stereomicroscope with GFP/RTF fluorescence was used.

### Quantitative real-time RT-PCR

Fresh leaf tissues (30 mg) was ground in liquid N_2_ and extracted with SV Total RNA Isolation System (Promega). RNA concentration was measured by Nanodrop (Thermo, USA). Reverse transcription (RT) reaction was performed by RevertAid First Strand cDNA synthesis kit (Fermentas) according to the manufacturer's instructions. All samples were run in triplicates. Primers 5′-AGTGGAGAGGGTGAAGGTGATG-3′ (forward) and 5′-TGATCTGGGTATCTTGAAAAGC-3′ (reverse) were used for GFP mRNA analysis. The *Nicotiana benthamiana* EF1 mRNS (GenBank accession number DQ321490) served as an internal control using primers 5′-TGGTGTCCTCAAGCCTGGTATGGTTG-3′ and 5′-ACGCTTGAGATCCTTAACCGCAACATTCTT-3′. Real-time PCR was carried out in Stratagene Mx300Pro machine, thermal cycling profile is described in Qu et al., 2007 [48].

### Histidine tagging

To tag the C-terminus of the 2b protein with hexahistidine (His-tag), 2b was amplified with the following oligonucleotides 5′-ATTGAGCTCGTAGTACAGAGTTCAGGG-3′ (forward) and 5′-GGATCCTCAGTGATGATGATGATGATGGAAAGCACCTTC-3′ (reverse) from pRs-2a777. This fragment was first cloned into pGEM-T easy vector than subcloned into pBin61s vector using SacI and BamHI restriction sites. To create histidine-tagged mutants, the tagged 2b C-terminus was subcloned into pBin61s containing the mutants 2b proteins using StuI-BamHI restriction sites.

### Protein analysis, SDS-PAGE, and immunoblotting

Protein extracts from *N. benthamiana* leaves were prepared from leaf samples (20 mg, fresh weight). Leaf discs were ground and homogenized in an ice-cold mortar in Laemmli solution, heated at 95°C for 5 min, and centrifuged (5 min at 10,000 g) to remove insoluble material. Aliquots of the supernatant (1 to 10 µ L) were separated by SDS-PAGE on 17, 5% gels. After electrophoresis, proteins were transferred to a Hybond-C membrane (GE Healthcare Bio-Sciences) and subjected to immunoblot analysis with Penta·His HRP Conjugate Kit following the manufacturer's instructions (Qiagen).

To detect the fluorescent proteins on SDS-PAGE, protein extracts were prepared from two discs leaf following the procedure described in [49]. Samples were separated on 12% gels. Fluorescent proteins were detected by illuminating the gel with UV lamp (UV Products, Blak-Ray B-100SP).

### Molecular modeling and graphics

The model structure of the full-length monomer CMV 2b protein was generated with I-TASSER [50] [51]. The model was built using the Rs-CMV 2b sequence. The NCBI/GenBank accession number is AJ517801. The main template was the X-ray structure of TAV 2b (PDB ID code: 2ZI0) to create the alpha helical regions (aa 1–69). Structure of the F1-ATPase from spinach chloroplasts (PDB ID: 1FX0) and structure of the Glia cell missing (GCM) transcription factor (PDB ID: 1ODH) were used to thread the predicted structure of the CMV 2b C-terminal domain (aa 65–110). The siRNA bound biologically active tetramer form was built with the Schrodinger Suite [52] molecular modeling software package. The completed tetramer siRNA-ribonucleoprotein complex was refined with energy minimization to eliminate the steric conflicts between the protein and RNA atoms. Molecular graphics were prepared using VMD version 1.9.1 [53].

## Supporting Information

Table S1 **Oligonucleotides used for creating the alanine-scanning mutants.**
(XLS)
